# Sagittal body alignment in a sitting position in children is not affected by the generalized joint hypermobility

**DOI:** 10.1038/s41598-021-93215-7

**Published:** 2021-07-02

**Authors:** Dariusz Czaprowski, Karolina Gwiazdowska-Czubak, Marcin Tyrakowski, Agnieszka Kędra

**Affiliations:** 1 Physiotherapy Unit, Department of Health Sciences, Medical University, Poznań, Poland; 2 Department of Health Sciences, Olsztyn University, Olsztyn, Poland; 3 Rehabilitation Clinic, Gruca Orthopaedic and Trauma Teaching Hospital, Otwock, Poland; 4 Department of Spine Disorders and Orthopaedics, The Centre of Postgraduate Medical Education in Warsaw, Warsaw, Poland; 5 Gruca Orthopaedic and Trauma Teaching Hospital, Otwock, Poland; 6 Faculty of Physical Education and Health, Jozef Pilsudski University of Physical Education in Warsaw, Biala Podlaska, Poland

**Keywords:** Paediatrics, Public health, Paediatric research

## Abstract

Back pain may be related to an improper sitting position. The aim of the study was to assess the sagittal curvatures of the spine in a sitting position in children with generalized joint hypermobility (GJH). The study included 302 children aged 8–14 years. The sagittal curvatures of the spine (sacral slope, lumbar lordosis, thoracic kyphosis with its lower and upper part) were assessed using the Saunders digital inclinometer. In order to assess GJH a 9-point Beighton scale was used. The study revealed no significant differences (p > 0.05) in sagittal curvatures of the spine in a relaxed sitting position between children with and without GJH. Regardless of the occurrence of GJH, kyphotic alignment of the spine was noted in a relaxed sitting. GJH does not affect the position of the trunk in a sagittal plane in a relaxed sitting position in children aged 8–14 years. A relaxed sitting position in children with and without GJH is characterized by a kyphotic position of the spine caused by an improper position of pelvis and lumbar segment of the spine.

## Introduction

Back pain (BP) typical of middle-aged individuals, occurs in young persons more often nowadays^1–5^. Kędra et al.^6^ revealed on a group of over 11,000 children and adolescents aged 10–19 years that BP occurred in nearly 75% of the respondents. In this study children aged 10–13 years the pain was most often located in the cervical region, while in adolescents aged 14–19 years—in the lower back^7^.

The prevalence of BP leads to increase in socio-economic costs connected with its diagnostics and therapy, absence from school/work or social reimbursements because of chronic disability^2,7^. Therefore, it is important to identify and modify the risk factors of BP that could reduce its occurrence or recurrence^2,7,8^.

Improper sitting position for a prolonged time is perceived as one of the factors leading to the development of BP^2,7,8^. Kędra et al.^7^ revealed that students who experienced BP spent 5 or more hours a day in a sitting position more often than students without pain (96.4% vs 63.6%, respectively). This study also revealed that the majority of students (67.4%) were not familiar with the rules of ergonomics used to modify a sitting position in order to reduce the intensity of BP or to prevent it^7^.

Generalized joint hypermobility (GJH) is diagnosed when the mobility of small and big joints is higher than normal for age, gender and race, when systemic diseases are excluded^9,10^. Generalized joint hypermobility may significantly affect body posture and the pattern of sitting due to decreased proprioception which leads to adoptions extreme positions in joints^9–15^. These factors together with increased activity of nociceptors may cause BP in children with GJH^9,11,12,14,15^. However, to date, no analysis of sagittal spinal curves in a relaxed sitting position in children and adolescents with GJH has been published. Therefore, because of the relation between a sitting position and BP as well as between BP and GJH, the aim of the study was to assess the sagittal curvatures of the spine in a relaxed sitting position in children with GJH.

## Materials and methods

### Material

The study included 302 children (145 girls, 157 boys) aged 8–14 years. The inclusion criteria were: age between 8 and 14 years, no injuries or pain in the musculoskeletal system within three months prior to the study and no other disorders that could affect the results.

The study was approved by the local ethical committee and a written consent was signed by a legal guardian of each child included in the study.

### Methods

In all the participants sagittal curvatures of the spine were assessed using the Saunders digital inclinometer (Baseline Digital Inclinometer, The Saunders Group Inc., Chaska, MN, USA)^16,17^.

Prior to the measurements a waterproof pen was used to mark the following anatomical landmarks on the back of each patient: the lumbosacral junction (L5–S1); the thoracolumbar junction (Th12–L1); the apex of thoracic kyphosis Th6–Th7; the cervicothoracic junction (C7–Th1)^18,19^. The measurements of the sagittal curvatures of the spine were performed in a standard relaxed sitting position. The child was sitting on a therapeutic table with adjustable height. This height of the table was set to keep the 90° angle in the hip and knee joints with feet fully and freely supported on the floor. Upper limbs were kept relaxed on both sides of the body. The participant was asked to “keep a natural relaxed sitting position”. Five seconds after that, sagittal curvatures of the spine were measured.

The measurements of the sagittal curvatures of the spine were performed according to the guidelines of the American Medical Association^16^. To measure sacral slope the inclinometer was calibrated to 0° in the horizontal plane and after that the angle at L5–S1 level was noted. In order to determine the angle of lumbar lordosis, the inclinometer was calibrated to 0° at L5–S1 level, and then the angle at Th12–L1 was assessed. Thoracic kyphosis was determined after calibrating the inclinometer to 0° at Th12–L1 level and then the angulation at C7–Th1 was measured^12,16,17^. Moreover, thoracic kyphosis was divided into its lower and upper part. The angle of the lower part of thoracic kyphosis was measured between the thoracolumbar junction (Th12–L1), where the device was calibrated to 0° and the Th6–Th7 junction. An upper part of thoracic kyphosis was measured from the Th6–T7 junction, where the device was calibrated to 0° up to the cervicothoracic junction (C7–Th1). According to the methodology all of the parameters were measured three times and the mean values of these three measurements were used in further statistical analysis^18,19^. The lordotic position of the examined segment was marked with “−” (minus).

Previous study revealed good repeatability and reliability of the measurements of sagittal curvatures of the spine with the Saunders inclinometer^18^.

All the measurements were taken by one researcher (Researcher #1) who was blinded to the diagnosis of GJH.

Researcher #2 assessed the GJH in all of the patients by use of a 9-point Beighton scale including: (1) passive hyperextension of the 5th finger of the hand over 90°; (2) passive apposition of the thumb to the forearm, (3) hyperextension of the elbow over 10°; (4) extension of the knee over 10°; (5) active forward bending of the trunk with the knees extended so that the palms of the hands rest flat on the floor^10^.

The tests regarding limbs were performed symmetrically on both sides. One point was given for the positive result of each part of the Beighton scale. Five points or more for girls and four points or more for boys were the cut-off values for the diagnosis of GJH^10^.

The study group was divided into two subgroups of girls and boys. In each of these subgroups the sagittal curvatures of the spine were compared between the individuals with and without GJH.

All the participants gave their written informed consent. The study was conducted in accordance with the Declaration of Helsinki, and the research was accepted by the Senate Scientific Research Ethics Commission of Olsztyn University, Poland (3/2020).

### Statistical analysis

Statistical analysis was performed by use of Statistica 7.1 software (StatSoft Poland) ^20^. Data distribution was verified by use of the Shapiro–Wilk test. The means were compared with Mann–Whitney U test and Student’s t-test. The level of significance was set at p < 0.05.

## Results

In both subgroups (girls and boys) no significant differences were found between the participants with and without GJH in the age, height, weight and body mass index (BMI) (p > 0.05) (Tables 1 and 2).

**Table 1:** Characteristics of girls with (GJH) and without (no-GJH) generalized joint hypermobility (n = 145).

	Girls GJH n = 38		Girls no-GJH n = 107	
	Mean	SD	Mean	SD
Age (years)	11.6	0.72	11.65	0.89
Height (m)	1.49	0.09	1.50	0.09
Weight (kg)	42.5	11.61	43.49	11.18
BMI (kg/m^2^)	18.7	4.09	18.96	3.74

**Table 2:** Characteristics of boys with (GJH) and without (no-GJH) generalized joint hypermobility (n = 157).

	Boys GJH n = 29		Boys no-GJH n = 128	
	Mean	SD	Mean	SD
Age (years)	11.0	1.53	11.2	1.44
Height (m)	1.46	0.13	1.46	0.11
Weight (kg)	41.1	15.36	40.52	13.45
BMI (kg/m^2^)	18.6	4.4	18.43	4.02

No statistically significant differences were found in all of the sagittal curvatures of the spine measured between girls with and without GJH (p > 0.05) (Table 3).

**Table 3:** Comparison of the sagittal curvatures of the spine in a relaxed sitting position between girls with (GJH) and without (no-GJH) generalized joint hypermobility.

	Girls GJH n = 38	Girls no-GJH n = 107	p
Sacral slope (°)	15.2° ± 8	14.4° ± 9.3	0.78
Lumbar lordosis (°)	18.4° ± 15.3	18.9° ± 10.7	0.82
Thoracic kyphosis (°)	36.3° ± 12.2	37.3° ± 10.4	0.67
Lower part of thoracic kyphosis (°)	14.5° ± 8.9	15.4° ± 9.1	0.63
Upper part of thoracic kyphosis (°)	21.5° ± 7.1	22.0° ± 7.9	0.72

Similarly, no significant differences in all the sagittal curvatures of the spine measured were noted between boys with and without GJH (Table 4).

**Table 4:** Comparison of the sagittal curvatures of the spine in a relaxed sitting position between boys with (GJH) and without (no-GJH) generalized joint hypermobility.

	Boys GJH n = 38	Boys no-GJH n = 107	p
Sacral slope (°)	10.8° ± 9.3	9.2° ± 8.2	0.31
Lumbar lordosis (°)	17.7° ± 10.7	14° ± 12.1	0.07
Thoracic kyphosis (°)	36.1° ± 13.5	36.8° ± 11.0	0.77
Lower part of thoracic kyphosis (°)	16.6° ± 10.9	15.0° ± 9.1	0.38
Upper part of thoracic kyphosis (°)	18.4° ± 10.4	21.7° ± 7.6	0.14

## Discussion

The aim of the study was to assess the influence of GJH on sagittal curvatures of the spine in children in a relaxed sitting position. To date, no such analysis has been published.

The study revealed no significant differences in all of the sagittal curvatures of the spine measured in a relaxed sitting position between girls with and without GJH as well as between boys with and without GJH. What we find interesting is the kyphotic position of the entire spine in all the subgroups (regardless gender and GJH). Thoracic kyphosis was within the normal values for a standing position (30°–40°) in all of the subgroups^16,19^. The referential value for sacral slope and lumbar lordosis in a standing position is (−) 15°–30° and (−) 30°–40°, respectively^16,19^. The study revealed that regardless of the GJH children aged 8–14 years adopted an improper kyphotic alignment of sacral slope and lumbar lordosis.

Children and adolescents spend significant amount of time in a sitting position, and this time increases with age^21^. Thus, the observed manner of sitting may significantly determine the occurrence of BP. It seems particularly significant in the case of children with GJH, since they present decreased ability to recognise the position of particular body segments with regard to one another. What is more, children with GJH present limited passive stabilisation of the spine connected with higher flexibility of connective tissues^9–15^. Therefore, appropriate educational programmes should be implemented in order to explain optimal manners of sitting aimed at reducing the risk of musculoskeletal system overloads and pain.

### Study limitations

The authors evaluated the individuals in a relaxed sitting position without back support. Therefore, a relaxed sitting position adopted by children during activities of daily living may differ from the position assessed in this study. However, this sitting position could be standardised and what is also important it enabled to obtain access to the examined curvatures of the spine.

The evaluation of sagittal alignment of the spine was performed shortly after a command “keep a natural relaxed sitting position” (5 s). We decided for such solution as we did not verify factors which could potentially influence the sagittal profile of the spine after longer period of time (e.g. subject’s motivation, strength of the trunk muscles). However, it would be interesting for future studies to analyse the influence of time on the sitting position and subsequently on the changes of the sagittal spinal alignment.

### Clinical relevance

The study was conducted by use on an objective reliable measurement tool—the Saunders digital inclinometer with previously verified technique^18^.

The analysis revealed that, regardless of GJH, children aged 8–14 years present a kyphotic position of the spine in a relaxed sitting position. It was a result of an improper position of the pelvis and the lumbar spine. Therefore, educational, prophylactic and therapeutic activities should be aimed mainly at improving the position of these two segments in sitting position. Moreover, the shape of the spine in the sagittal plane in a relaxed sitting position did not differentiate children with and without GJH. As GJH may be one of the factors leading to BP, it is important to apply specific tests for GJH which will help to diagnose it in clinical practice. It is important as the rules of physiotherapeutic procedures regarding individuals with GJH differ from the standard exercises. Firstly, children with GJH should not perform stretching exercises since they may increase lumbosacral pain^22,23^. On the other hand, the aim of the exercises addressed to children with GJH is improving the stability of lumbo–pelvic–hip complex and proprioception as well as on education in order to avoiding the adoption of extreme position in the joints during activities of daily living^9,14,23,24^.

The evaluation of lower and upper part of the thoracic kyphosis is an additional advantage of the study. It revealed that both the lower and the upper part of the thoracic spine were in a normal kyphotic position. Regardless of the occurrence of GJH, higher values of kyphosis were noted for an upper part of the thoracic segment, what indicates the tendency for bigger inclination of this part of the thoracic spine, which may subsequently lead to a head protraction. However, this observation needs further research that would include the assessment of the position of head and cervical segment of the spine.

## Conclusions

1. Generalized joint hypermobility does not affect the position of the trunk in a sagittal plane in a relaxed sitting position in children aged 8–14 years.
2. A relaxed sitting position in children with and without generalized joint hypermobility is characterized by a kyphotic alignment of the whole spine caused by an improper position of pelvis and lumbar segment of the spine.
3. The sagittal curves of the spine in sitting position should not be used to diagnose generalized joint hypermobility. Thus the tests specific for generalized joint hypermobility should be applied in clinical practice.
